# Supplementary material for: Deep Eutectic Liquids as a Topical Vehicle for Tadalafil: Characterisation and Potential Wound Healing and Antimicrobial Activity
Source: Molecules. 2023 Mar 6;28(5):2402. doi: 10.3390/molecules28052402 (PMC10005105; doi:10.3390/molecules28052402)
Supplement: Supplementary file 1 [file molecules-28-02402-s001.zip › molecules-2214506-supplementary.pdf]

Deep Eutectic liquids as a Topical Vehicle of Tadalafil: Characterization and Potential Wound Healing and Antimicrobial Activity

Bayan Alkhawaja[1], Faisal Al-Akayleh[2*], Ashraf Al-Khateeb[2], Jehad Nasiraldeen[3], Bayan Y. Ghanim[4], Albert Bolhuis[5], Nisreen Jaber[6], Mayyas Al-Remawi[2], Nidal A. Qinna[4]

[1]Department of Pharmaceutical Medicinal Chemistry and Pharmacognosy, Faculty of Pharmacy and Medical Sciences, University of Petra, Amman 11196, Jordan

[2]Department of Pharmaceutics and Pharmaceutical Technology, Faculty of Pharmacy and Medical Sciences, Petra University, Amman 11196, Jordan

[3]Depratment of Pharmaceutical Sciences, Faculty of Pharmacy, Zarqa University, Zarqa 13110, Jordan

[4] University of Petra Pharmaceutical Center, Faculty of Pharmacy and Medical Sciences, Petra University, Amman 11196, Jordan

[5] Department of Life Sciences, University of Bath, Claverton Down, BA2 7AY, Bath, UK

[6] Faculty of Pharmacy, Al-Zaytoonah University of Jordan, Amman 11733, Jordan

*Corresponding author’s email: falakayleh@uop.edu.jo (F. Al-Akayleh)

University of Petra, Amman 11196, Jordan

Supplementary results

1- Linearity of the HPLC method utilized for the analysis of TDF and LDC

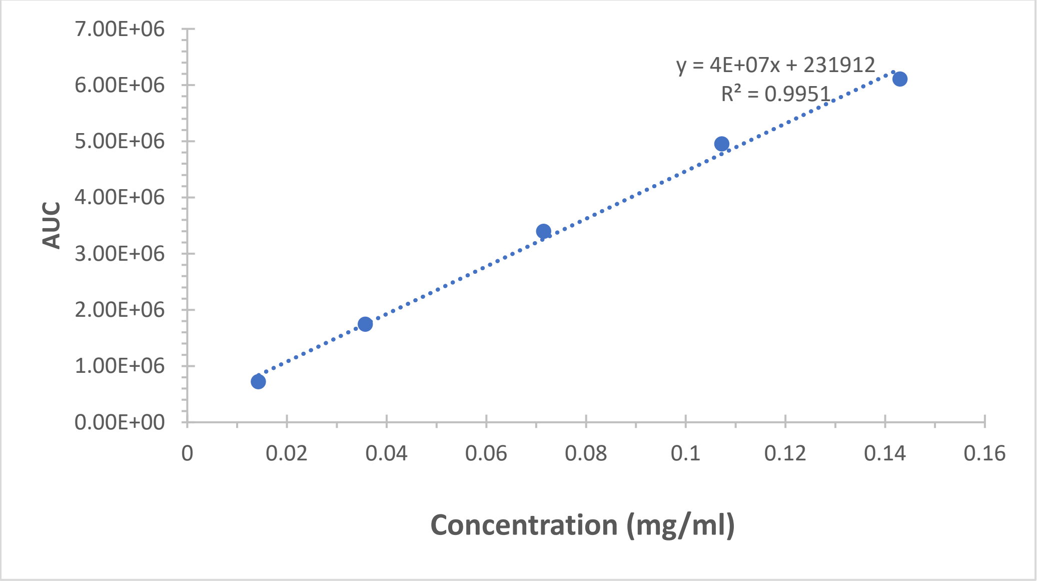

Figure S1. Standard curve of TDF.

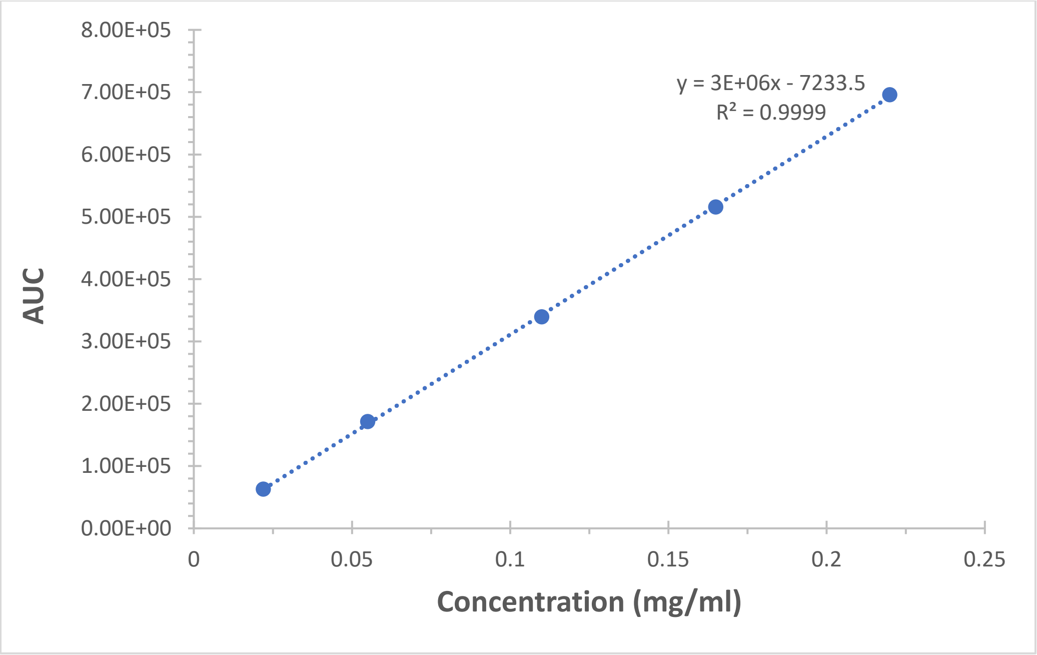

Figure S2. Standard curve of LDC.

2- NMR of the individual components

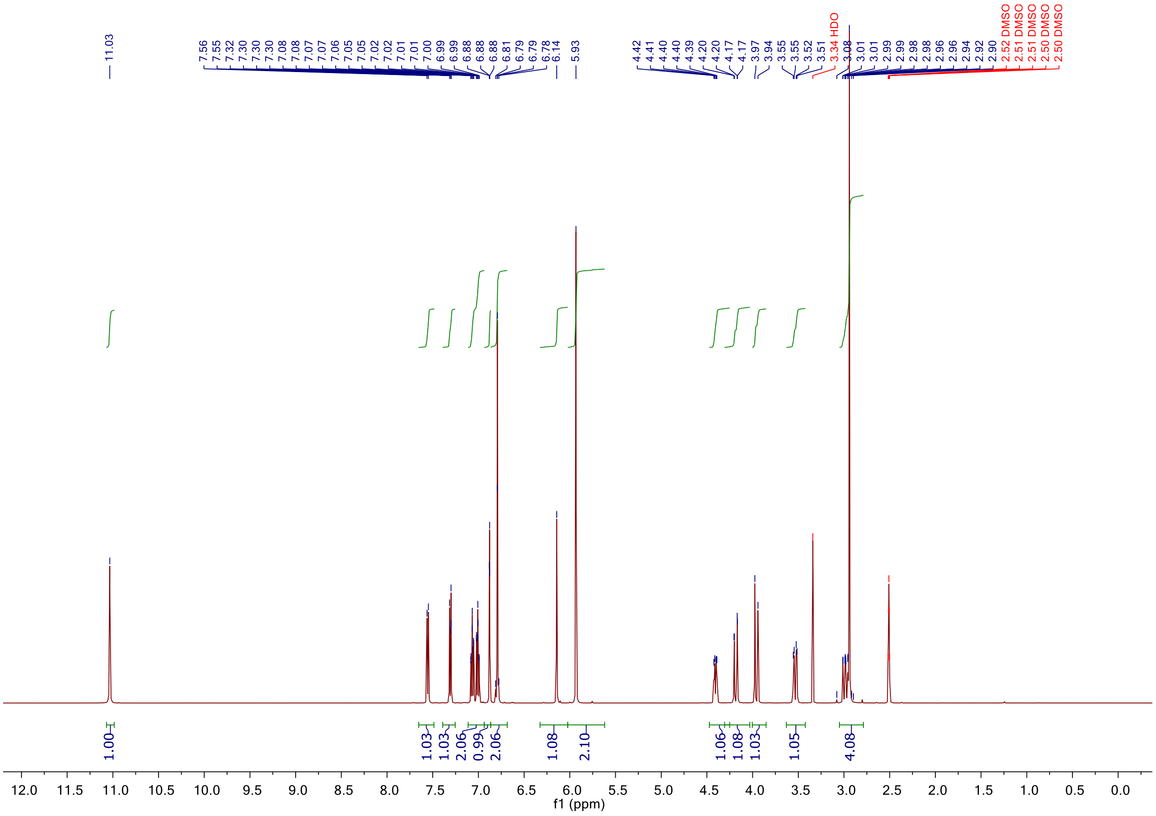

Figure S3. 1H NMR spectrum of TDF.

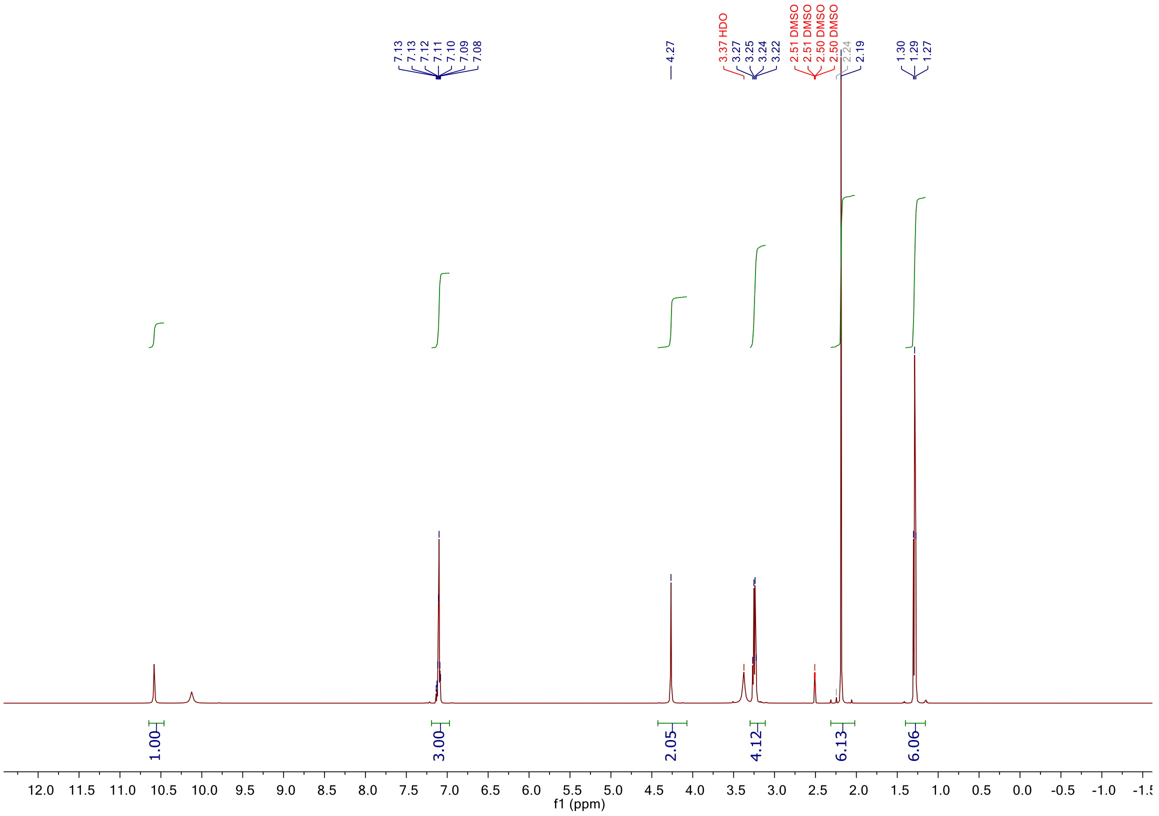

Figure S4. 1H NMR spectrum of LDC.
